# Quantitative trait loci pyramiding for fruit quality traits in tomato

**DOI:** 10.1007/s11032-012-9763-2

**Published:** 2012-06-28

**Authors:** Adriana Sacco, Antonio Di Matteo, Nadia Lombardi, Nikita Trotta, Biancavaleria Punzo, Angela Mari, Amalia Barone

**Affiliations:** 1 Department of Soil, Plant, Environmental and Animal Sciences, University of Naples Federico II, Portici, Italy; 2 CRA-ORT, Italian Agricultural Research Council, Research Centre for Vegetable Crops, Pontecagnano, Italy; 3 CNR-ISA, Institute for Food Science, Avellino, Italy; 4 Present Address: Department of Pharmaceutical and Biomedical Sciences, University of Salerno, Salerno, Italy

**Keywords:** *Solanum lycopersicum*, *S. pennellii* introgression lines, Molecular markers, Ascorbic acid, Phenols, Soluble solids

## Abstract

Fruit quality is a major focus for most conventional and innovative tomato breeding strategies, with particular attention being paid to fruit antioxidant compounds. Tomatoes represent a major contribution to dietary nutrition worldwide and a reservoir of diverse antioxidant molecules. In a previous study, we identified two *Solanum pennellii* introgression lines (IL7-3 and IL12-4) harbouring quantitative trait loci (QTL) that increase the content of ascorbic acid (AsA), phenols and soluble solids (degrees Brix; °Bx) in tomato fruit. The purpose of the present work was to pyramid into cultivated varieties the selected QTL for enhanced antioxidant and °Bx content. To better understand the genetic architecture of each QTL, the two ILs were crossed to the recurrent parent M82 (ILH7-3 and ILH12-4) and between them (ILH7-3+12-4). F1 hybrids (ILH7-3+12-4) were then selfed up to obtain F3 progenies in order to stabilize the favourable traits at the homozygous condition. Species-specific molecular markers were identified for each introgressed region and allowed us to select four F2 genotypes carrying both introgressions at the homozygous condition. The F3 double homozygous plants displayed AsA, total phenols and °Bx content significantly higher than M82. Therefore, they may represent suitable genetic material for breeding schemes aiming to increase antioxidant content in tomato fruit.

Consumers are paying ever-increasing attention to the health and nutritional aspects of horticultural products, such as vitamin content, mineral elements and antioxidants (Causse et al. 2010). Fresh fruit and vegetables have long been regarded as having considerable health benefits, due in particular to their antioxidant content, which can protect the human body against cellular oxidation reactions, decreasing the risk of some common diseases such as cardiovascular events, cancer and other age-related degenerative diseases (Demmig-Adams and Adams 2002). These benefits have stimulated researchers to investigate the antioxidant content of fruit and vegetables, and to highlight the molecular mechanisms underlying their synthesis and accumulation in plants.

Tomatoes represent a major contribution to nutrition worldwide and a reservoir of diverse antioxidant molecules, such as ascorbic acid (AsA or vitamin C), vitamin E, carotenoids, flavonoids and phenolic acids. In addition, to obtain in-depth knowledge of genetic regulation of nutritional content compounds in fruits, tomato also represents an excellent model system for both basic and applied research for many reasons, including ease of growth in a wide range of environments, a short life-cycle, and well-developed genetic and genomic tools (Foolad 2007; Barone et al. 2008). Consequently, fruit quality in terms of both nutritional and organoleptic quality [flavour, antioxidant, degrees Brix (°Bx) content, etc.] has become a major focus of most traditional and innovative tomato breeding approaches. Such traits usually exhibit quantitative variation controlled by several genes, and influenced by environmental conditions (Schauer et al. 2008). Molecular markers allow the dissection of such quantitative traits into discrete quantitative trait loci (QTL), which can be located on the tomato genetic map (Frary et al. 2005). Moreover, the development of molecular marker-assisted selection (MAS) has enabled the construction of a population of introgression lines (ILs) (Eshed and Zamir 1995), where each IL includes single marker-defined introgressed genomic regions from the wild green-fruited species *Solanum pennellii* into the genomic background of the cultivated *S. lycopersicum* inbred variety M82. Overall, the IL population provides complete coverage of the wild-species genome and allows the reservoir of wild genes to be investigated. In particular, *S. pennellii* introgression lines were shown to be powerful material for dissecting plant yield and fruit quality (Gur and Zamir 2004; Rousseaux et al. 2005; Stevens et al. 2007). In addition, this population has already been used for mapping candidate genes and QTL for carotenoids, fruit weight and composition in sugars and acids, antioxidant compounds, volatile aromas and various metabolites (Lippman et al. 2007).

In our laboratory, two *S. pennellii* ILs (IL7-3 and IL12-4) were identified for producing higher antioxidant and soluble solids fruit content in comparison with the *S. lycopersicum* variety M82, following three-year screening under greenhouse conditions. In particular, one QTL for high AsA content was identified in the introgressed region 12-4 (Di Matteo et al. 2010a) and one QTL for high AsA, phenols and °Bx content in the introgressed region 7-3 (Di Matteo et al. 2010b). The aim of this study was to produce new genetic materials with combined high fruit antioxidant and °Bx content. For this purpose, we pyramided these QTL in the genetic background of the cultivated processing tomato M82 with the aid of introgression-specific molecular markers by selecting markers suitable for distinguishing the wild genome of *S. pennellii* from the cultivated *S. lycopersicum*.

Seeds from IL12-4 (LA4102), IL 7-3 (LA4066), and their parental line M82 (LA3475) were kindly provided by The Tomato Genetics Resource Centre (TGRC) (http://tgrc.ucdavis.edu/). Crosses between these lines were made in order to assay the interaction among QTL carried by introgressions 7-3 and 12-4. In particular, to better understand the genetic architecture of each QTL, the two ILs were crossed to the recurrent parent M82 (ILH7-3 and ILH12-4) and between them (ILH7-3+12-4). Markers that allowed differentiation between the wild and cultivated alleles were selected by exploring the Sol Genomics Network database (www.solgenomics.net/search/markers). To this end, markers on chromosomes 7 and 12 were sought in the chromosomal regions extending from 39 to 64 cM and from 74 to 120 cM for IL7-3 and IL12-4, respectively. Eighteen markers tested for targeting 7-3 and 12-4 introgressions showed polymorphism between parental lines. In particular, for 17 markers tested, PCR fragments amplified from each parental genotype were sequenced and restriction enzymes that discriminated genotypes were selected. Only in one case (AT3g17000) was the difference due to an insertion/deletion and no sequencing of PCR fragments was required. In total, four and three markers for introgression regions 7-3 and 12-4 were chosen, respectively (Table 1). These mapped approximately at the top, middle and bottom of each region. These markers were used to validate hybrid genotypes as harbouring alleles from both parents.

**Table 1:** Selected markers for both introgression regions 7-3 and 12-4, which were used for screening ILH7-3, ILH12-4, ILH7-3+12-4 and IL7-3+12-4

Introgression region	Marker	Position (cM)	PCR product (bp)	Restriction enzyme	Restriction fragment size^a^ (bp)	
					*S. pennellii*	*S. lycopersicum*
7-3	C2_At2g20830	43.00	550	*Bgl*II	450 + 100	380 + 100 + 70
7-3	C2_At1g17200	46.00	700	*Hae*III	500 + 180	500 + 200
7-3	C2_At1g53670	54.00	1,350	*Taq*I	700 + 400	1000 + 100
7-3	U220926	63.70	370	*Alu*I	230 + 140	370
12-4	T0801	74.00	600	*Rsa*I	340 + 180 + 60	520 + 60
12-4	At4g31150	96.00	400	*Eco*RV	400	250 + 150
12-4	AT3g17000	115.00	1,300/1,100	–		

cM distance refers to the Tomato-EXPEN 2000 map available at the Sol genomics network, http://solgenomics.net

^a^Only the digestion pattern that allowed distinction of *S. pennellii* from *S. lycopersicum* is reported

The hybrids and their parental lines (five plants/genotype) were then grown in a cold greenhouse according to a completely randomized design. Fruits were collected when 75 % were full-sized and red-ripe, softening had increased and the inside of the columella was completely red. Chemical properties of 10–20 red-ripe fruit per genotype were assessed. A modified version of the enzymatic method described by Stevens et al. (2006) was used for reduced AsA determination. Briefly, tomato fruits were ground in liquid nitrogen, and 500 mg of powder was homogenized with 30 μL of ice-cold 6 % trichloroacetic acid (TCA). Samples were centrifuged for 20 min at 20,000*g* at 4 °C. 20 μL 0.4 M phosphate buffer and 10 μL H_2_O were added to a 20 μL aliquot of the supernatant followed by the addition of 80 μL of color reagent. The color reagent was made up as follows: solution A = 31 % orthophosphoric acid, 4.6 % (w/v) TCA and 0.6 % (w/v) iron chloride (FeCl_3_); solution B = 4 % 2,2-dipyridyl (w/v; made up in 70 % ethanol). Solutions A and B were mixed 2.75 parts A to 1 part B. After incubation at 37 °C for 40 min, the absorbance was read at 550 nm using a NanoPhotometer™ (Implen).

Folin-Ciocalteu’s procedure (Singleton and Rossi 1965) was used for phenol quantification, and a portable refractometer ATAGO Model ATC-1 for measuring soluble solids. The AsA concentration was expressed in μmol g^−1^ fresh weight (FW) according to the standard curve A_525_ = 3.6593 × μmol AsA, designed over a dynamic range of 0–0.7 μmol AsA (*R* ^2^ = 0.9982). Concentrations were then converted into mg (100 g FW)^−1^. Total phenolic concentration was expressed in terms of mg of gallic acid equivalents (GAEs) (100 g FW)^−1^, based on a gallic acid standard curve designed over a dynamic range of 0–125, which is A_760_ = (0.0234 × μg gallic acid) − 0.0776 (*R* ^2^ = 0.995). Soluble solids content was expressed on a °Bx scale. Data collected were analyzed by means of parametric tests by using SPSS (Statistical Package for Social Sciences) Package 6 version 15.0. Significance was determined by comparing mean values of ILs and ILHs to the control M82 through a factorial analysis of variance (one-way ANOVA) with LSD post hoc test at a significance level of 0.05.

The AsA fruit content of plants heterozygous for introgressions 7-3 (ILH7-3) and 12-4 (ILH12-4) was no different from the control, leading us to hypothesize the recessive inheritance of both QTL controlling AsA in the wild species *S. pennellii* (Fig. 1). By contrast, ILH7-3+12-4 plants displayed a significantly higher AsA content (+39 %) with respect to M82 control, thus suggesting a positive epistatic interaction between the AsA QTL mapping to the two introgressed regions (7-3 and 12-4). Looking at the total phenol content of the ILH7-3+12-4 fruits we can assume the recessive inheritance for the phenol QTL with no more speculation, since the data for ILH7-3 were missing because not enough fruit samples were available for the statistical analyses. Finally, °Bx contents of ILH7-3+12-4 and ILH7-3 were significantly higher (+22 %) than the control leading us to suppose the dominant inheritance of this trait.

**Fig. 1:**
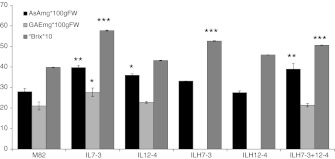
Ascorbic acid, total phenols and °Bx content in red-ripe fruit from the tomato IL7-3, IL12-4, ILH7-3, ILH12-4, ILH7-3+12-4 genotypes and the parental line M82. The mean values (±SE) are reported for at least three plant replicates grown under greenhouse conditions in 2009. *Asterisks* indicate statistically significant differences compared to M82 (*P* < 0.05)

In order to pyramid these QTL in new genetic materials, the QTL under study must be stabilized at the homozygous condition. For this purpose, the ILH7-3+12-4 genotypes were selfed to obtain F2 genotypes. Indeed, genotypes obtained by intercrossing the two ILs IL7-3 and IL12-4 are homozygous in all regions that carry the M82 genetic background, but heterozygous only in the two introgression regions. Therefore, by selfing ILH7-3+12-4 the segregation of both introgressed heterozygous regions will occur and 1/16 of genotypes simultaneously homozygous in these two regions is expected. Sixty-eight F2 genotypes obtained by selfing ILH7-3+12-4 were screened by the seven markers previously chosen, which allowed selection of four genotypes carrying both introgressions 7-3 and 12-4 at the homozygous condition (IL7-3+12-4). This number is consistent with the expected value of 4.25 genotypes out of a total of 68 analyzed. The double homozygous plants obtained, which were stable in all their genomic regions, were then selfed and F3 families (10 plants/family in three replicates) were field-grown in southern Italy.

As shown in Fig. 2, the IL7-3+12-4 displayed significantly higher AsA, total phenol and °Bx content with respect to M82. The AsA content of the two ILH7-3 and ILH12-4 was confirmed to be no different from M82 under field conditions also, thus strengthening the recessive inheritance of the AsA QTL. As for AsA QTL, our hypothesis on the inheritance mode of the other two QTL for phenol and °Bx was supported by the results obtained in the field. The ILHs (ILH7-3, ILH12-4 and ILH7-3+12-4) showed a similar phenol content to the control and a significantly higher °Bx value than M82, confirming the recessive and dominant inheritance of phenols and °Bx QTL, respectively. Comprehensively, despite the values of the different metabolites measured depending on the environment and growth season, as often reported in the literature (Rousseaux et al. 2005; Schauer et al. 2008), in our study data collected from the greenhouse and field trials confirmed the presence of the QTL under investigation.

**Fig. 2:**
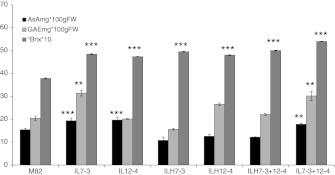
Ascorbic acid, total phenols and °Bx content in red-ripe fruit from the tomato IL7-3, IL12-4, ILH7-3, ILH12-4, ILH7-3+12-4, IL7-3+12-4 genotypes and the parental line M82. The mean values (±SE) are reported for at least seven replicates of plants field-grown in 2010. *Asterisks* indicate statistically significant differences compared to M82 (*P* < 0.05)

Finally, since IL7-3 has been reported as carrying negative QTL for yield (Eshed and Zamir 1995), total plant yield (kg/plant) was also assayed for all double homozygous genotypes, hybrid genotypes and their cultivated parental line. As expected, the worst performance in terms of production was shown by IL7-3 (Fig. 3), whereas all the ILHs showed a production which was similar to, or higher than, the control M82, as often reported in the literature (Gur and Zamir 2004; Lippman et al. 2007). The IL7-3+12-4 displayed an intermediate total plant yield (3.6 kg/plant) between IL7-3 (1 kg/plant) and ILH7-3+12-4 (7.4 kg/plant). In particular, since the yield of IL7-3+12-4 was significantly higher than IL7-3, the introgression 12-4 may well have had a compensating effect on the negative IL7-3 yield QTL.

**Fig. 3:**
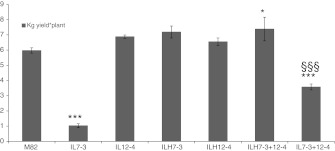
Total plant yield (kg/plant) of IL7-3, IL12-4, ILH7-3, ILH12-4, ILH7-3+12-4, IL7-3+12-4 genotypes and the parental line M82. The mean values (±SE) are reported for at least seven plant replicates grown in the open field in 2010. *Asterisks* indicate statistically significant differences compared to M82 (*P* < 0.05), *section symbol* indicates statistically significant differences compared to IL7-3 (*P* < 0.05)

In conclusion, the ILs were tested under homozygote and heterozygote conditions, facilitating determination of the inheritance mode of each QTL for AsA, total phenol and °Bx content. These ILs were used to generate hybrid genotypes with increased °Bx content and a yield similar (ILH7-3 and ILH12-4) or higher (ILH7-3+12-4) than M82. Moreover, we pyramided the four QTL for fruit quality into a unique background (IL7-3+12-4) with no dramatic yield decrease compared to IL7-3, thus obtaining suitable genetic material for a breeding scheme aiming to increase antioxidant content in tomato fruit. All these genetic materials could be of extreme interest to seed companies and the plant breeding industry, which could use them as a starting point to generate new hybrids or improved varieties.

Indeed, IL heterosis has already progressed beyond scientific publications into practical use in agriculture. A QTL pyramiding study with three independent yield-promoting introgressions resulted in a hybrid with 50 % higher yields than leading commercial varieties in multiple environments and irrigation regimes (Gur et al. 2011). Moreover, introgressions originating from *S. pennellii* were introduced into lines of processing tomato, and the resulting hybrid AB2 is presently a leading variety in California (http://www.ptab.org/ranking9.htm), which is the largest producer of processing tomatoes in the world. These real-world applications illustrate how ‘exotic’ alleles from interspecific diversity can enrich the genetic basis of cultivated plants to improve productivity (Lippman et al. 2007). In our study, the IL7-3+12-4 showed increased AsA (+15 %), phenol content (+47 %), and °Bx (+43 %) but decreased yield (−40 %) with respect to M82. These results confirmed that crosses between ILs give a chance of obtaining useful material for breeding, even though in our case a better definition of the “useful” introgression region 7-3 is required, as also suggested for rice by Ashikari and Matsuoka (2006). This could be achieved by chromosome recombination between the IL7-3+12-4 progeny and the recurrent parent followed by MAS that might lead to obtaining different sub-lines of the region carrying desirable and undesirable traits. Indeed, the information deriving from the complete sequencing of the tomato genome (Tomato Genome Consortium 2012) will shortly facilitate identification and mapping of candidate genes for antioxidant fruit content and plant production in the introgression region 7-3. Therefore, further investigation will facilitate the dissection of the introgressed region into reduced sub-lines, helping select those that decouple positive from negative traits.
